# Cardiovascular disease risk factor burden and cognition: Implications of ethnic diversity within the Hispanic Community Health Study/Study of Latinos

**DOI:** 10.1371/journal.pone.0215378

**Published:** 2019-04-22

**Authors:** Melissa Lamar, Ramon A. Durazo-Arvizu, Shruti Sachdeva, Amber Pirzada, Krista M. Perreira, Tatjana Rundek, Linda C. Gallo, Ellen Grober, Charles DeCarli, Richard B. Lipton, Wassim Tarraf, Hector M. González, Martha L. Daviglus

**Affiliations:** 1 Rush Alzheimer’s Disease Center and the Department of Behavioral Sciences, Rush University Medical Center, Chicago, Illinois, United States of America; 2 Institute for Minority Health Research, University of Illinois at Chicago, Chicago, Illinois, United States of America; 3 Department of Medicine, University of Illinois at Chicago, Chicago, Illinois, United States of America; 4 Department of Public Health Sciences, Loyola University, Chicago, Illinois, United States of America; 5 Department of Public Policy, University of North Carolina at Chapel Hill, Chapel Hill, North Carolina, United States of America; 6 Department of Neurology, University of Miami Health System, Miami, Florida, United States of America; 7 Department of Psychology, San Diego State University, San Diego, California, United States of America; 8 Department of Neurology, Albert Einstein College of Medicine, New York, New York, United States of America; 9 Department of Neurology, University of California Davis Health System, Sacramento, California, United States of America; 10 Institute of Gerontology, Wayne State University, Detroit, Michigan, United States of America; 11 Department of Neurosciences and Shiley-Marcos Alzheimer’s Disease Research Center, University of California San Diego, San Diego, California, United States of America; Case Western Reserve University, UNITED STATES

## Abstract

**Objective:**

Hispanics/Latinos have some of the highest prevalence rates for cardiovascular disease risk factors, but stark differences exist by self-reported background. Cardiovascular disease risk factors negatively impact cognition in Hispanics/Latinos; less is known about these relationships by Hispanic/Latino backgrounds. We investigated cognitive associations with cardiovascular disease risk factor burden in a diverse cohort, the Hispanic Community Health Study/Study of Latinos.

**Methods:**

Baseline data from this observational study of cardiovascular disease and its antecedents was collected from 2008–2011. We included 7,121 participants 45–74 years old from Central American, Cuban, Dominican, Mexican, Puerto Rican, or South American backgrounds. Dichotomous indicators for hypertension, diabetes, hypercholesterolemia, obesity, and smoking were evaluated and totaled, with participants grouped by lowest (0–2), middle (3) or highest (4–5) burden. Cognitive testing included the Brief Spanish English Verbal Learning Test, letter fluency, and digit symbol substitution.

**Results:**

In separate fully-adjusted linear regression models, lower fluency and digit symbol substitution performance were restricted to the highest compared to the lowest burden group; whereas the middle burden group displayed impaired memory performance compared to the lowest burden group (p-values≤0.05). Background interacted with burden for learning and memory performance. That is, the association of burden level (i.e., lowest, middle, or highest) with cognitive performance was modified by background (e.g., Mexicans vs Cuban).

**Conclusions:**

Hispanics/Latinos with higher levels of cardiovascular disease risk factor burden displayed lower levels of cognitive performance, with learning and memory performance modified by background.

## Introduction

Approximately 12% of older Hispanics/Latinos are diagnosed with Alzheimer’s disease (AD). While this is the highest proportion among US ethnic groups [1], differences in incidence rates of dementia including AD have also been reported by Hispanic/Latino background [2]. For example, Hispanics/Latinos from a Mexican background have a reported 0.8% annual incidence of dementia while Hispanics/Latinos from a Caribbean background have a 2.3%-5.3% annual incidence of dementia [3]. These distinctions may be due, in part, to distinctions in cumulative cardiovascular disease risk factor (CVD-RF) burden including the presence of the major and readily measurable risk factors of hypertension, diabetes, hypercholesterolemia, obesity, and smoking [4–6]. In fact, studies comparing racially or ethnically diverse populations consistently report that higher cumulative CVD-RF burden is associated with lower cognitive performance [7–10]. Furthermore, it is well-known that baseline CVD-RF burden increases the risk of later cognitive impairment and dementia in non-Hispanic Whites and Blacks [11–13] with increasing support in Hispanics/Latinos [7, 14]. Less work has enumerated the cognitive correlates of cumulative CVD-RF burden in Hispanics/Latinos by self-reported background despite stark differences in rates of CVD-RF burden.

While marked variation in rates of individual and cumulative CVD-RF burden exist based on Hispanic/Latino background, less is known about how this variation by background may impact known associations between CVD-RFs and cognition [2]. Puerto Ricans have the highest rates of both obesity and smoking [6, 15]. Furthermore, cumulative CVD-RF burden including the presence of insulin resistance, hyperlipidemia, hypertension, and obesity is highest in Puerto Ricans and lowest in South Americans [16]. A recent study [17] reported that Hispanics/Latinos with a Puerto Rican background display lower cognitive scores on all but a mental processing speed test when compared to Hispanics/Latinos with a Mexican background. While these investigators discussed the possibility that distinct CVD-RF profiles may underlie specific associations between background and cognition, this potential effect modifier was not explored. Given that a complete lack of CVD-RF burden (i.e., no burden) in mid- to late-life Hispanics/Latinos is rare, ranging from 1.8% in Mexican women to 0.6% in Cuban men [5], investigating cumulative CVD-RF burden and cognition by self-reported background may not only point toward a possible mechanism responsible for background distinctions in cognition, but also inform more specific clinical management as it relates to the diversity within the Hispanic/Latino community.

We investigated cumulative CVD-RF burden as it relates to cognition with a focus on major and readily measurable CVD-RFs [5] known to negatively and differentially impact Hispanics/Latinos by self-reported background [6, 15]. Thus, we focused on cumulative CVD-RF burden of hypertension, diabetes, hypercholesterolemia, obesity, and smoking in Hispanic/Latino self-reported backgrounds of Mexican, Cuban, Puerto Rican, Dominican, Central and South American. Using data from the largest cohort study of Hispanics/Latinos in the US we investigated differences in cognitive test scores of episodic learning and memory, fluency, and mental processing speed as a function of level of CVD-RF burden. First, we hypothesized that there would be a negative relationship between cumulative CVD-RF burden and cognition in Hispanics/Latinos generally, i.e., the highest CVD-RF burden group would be associated with the lowest cognitive performance when compared to all other burden groups, regardless of background. Additionally, we hypothesized that the relationship between CVD-RF burden group and cognition would vary by Hispanic/Latino background. Given that Puerto Ricans have the highest levels of overall cumulative CVD-RF burden while individuals of Mexican and Cuban backgrounds have some of the lowest, we specifically hypothesized that Puerto Ricans would show the expected association of higher CVD-RF burden to lower cognitive performance while individuals of Mexican or Cuban background would not.

## Materials and methods

The HCHS/SOL is a population-based, probability sample prospective cohort study of 16,415 Hispanics/Latinos aged 18–74 years from Chicago, IL, Miami, FL, Bronx, NY, and San Diego, CA [18]. In 2008–2011 the HCHS/SOL sampled households in these four US cities using a stratified 2-stage area probability sample design. This design oversampled certain areas to increase the likelihood that a selected address would yield a Hispanic/Latino household, and oversampled those ages 45–74 years to facilitate examination of target outcomes [18]. The baseline examination (2008–2011) [19] was conducted by trained professionals in a single visit at each city’s designated study site and consisted of biological, behavioral and socio-demographic assessments as well as a review and scanning of all prescription medications [19]. Cognitive testing was also conducted during this baseline examination, but only for individuals 45 years and older. The cohort includes participants who self-identified as having a Central American, Cuban, Dominican, Mexican, Puerto Rican, or South American background and oversampled persons ages 45–74. This study was approved by the Institutional Review Boards at each site, with the University of Illinois at Chicago Office for the Protection of Research Subjects IRB #3 providing the approval for our specific study. All participants gave written informed consent.

### Participants

Men and women ages ≥45 years with complete CVD-RF and cognitive information contributed to this analysis. We excluded participants who self-reported acute stroke (n = 188), substance abuse (n = 347), or were found to have psychotropic medication use including anti-anxiolytics, antidepressants, and antipsychotics based on medication review at study visit that entailed visual inspection and scanning of prescriptions (n = 1,051), or who were missing data on any covariates (n = 516). This resulted in 7,121 participants.

### Determination of cumulative CVD-RF burden

This study focused on the presence of the major and readily measurable risk factors hypertension, diabetes, hypercholesterolemia, obesity, and smoking [4–6]. Criteria for defining each CVD-RF for individual participants are outlined below. For those involving blood levels, 12 hour fasting blood draws were conducted by trained and certified clinic staff as part of a larger blood biometrics data collection soon after participant arrival for their baseline visit, stored at -20°C, and shipped every week for analysis. All field center procedures and laboratory protocols for blood levels are published online and available from: http://www2.cscc.unc.edu/hchs/manuals-forms.

*Hypertension*: determined using the definition implemented in the NHANES [20]. Thus, blood pressure was measured on the right arm by trained and certified clinic staff using an OMRON HEM-907 XL (Omron Healthcare, Inc., Lake Forest, IL) automatic sphygmomanometer with the participant in a seated position and the arm resting. Three readings were obtained at 1-minute intervals following a 5-minute rest period with the average of the three systolic blood pressure readings used as a covariate. If systolic or diastolic blood pressure was greater than or equal to 140/90 or if antihypertensive medications were provided during the medication review at the study visit, the participant was deemed hypertensive. *Impaired Fasting Glucose (PreDiabetes) or Diabetes*: determined using the criteria set forth by the American Diabetes Association [21] that included at least one of the following values, fasting glucose≥100mg/dL or hemoglobin A1c≥5.7%. Medications use for diabetes, verified at the study visit as previously described, was also considered in the determination of diabetes; *Hypercholesterolemia*: fasting total cholesterol ≥200mg/dL and/or use of cholesterol lowering medications verified at the study visit as described above; *Overweight or Obese*: BMI≥25kg/m^2^; *Smoking*: positive self-report of current smoking only.

Dichotomous indicators for the five CVD-RFs were summed to determine total burden. Total burden was used to classify individuals into CVD-RF burden groups. More specifically, the lowest-burden group consisted of participants with 0 or 2 CVD-RFs, the middle-burden group of participants with exactly 3 CVD-RFs and the highest-burden group consisted of participants with 4 or 5 CVD-RFs. These distinctions were made, in part, by the sample distribution, e.g., very few participants had 0 or 5 CVD-RFs (2.0% and 2.5%, respectively) while nearly equal numbers had 3 versus 1 to 2 CVD-RFs, etc.

Our choice to use dichotomous indicators of the CVD-RFs outlined above in order to calculate burden as opposed to employing continuous variables of risk or previously published cumulative risk scores stems from several factors. One, a large number of HCHS/SOL publications have documented the prevalence and incidents rates of each of the five major and measurable CVD-RFs outlined above on Hispanics/Latinos generally and by self-reported background specifically [5, 6, 15, 16, 22]; thus, adequate documentation for their importance and applicability to this population exists. Two, these five CVD-RFs may be easily queried during clinical interviews, providing a relatively quick means to evaluate an analogous self-reported cumulative CVD-RF burden score. Furthermore, previously published cumulative burden scores may exclude more relevant (e.g., obesity) aspects of cardiovascular disease risk, morbidity, and/or mortality in Hispanics/Latinos when compared to non-Hispanic Whites [2].

### Cognitive testing

Tests were administered in the participants’ preferred language (Spanish/English) during face-to-face interviews by staff trained/supervised by doctorate-level, licensed, clinical neuropsychologists. While only four tests of cognition were administered, they assessed important outcomes associated with aging including learning, memory, and attention/executive functioning. The Brief Spanish English Verbal Learning Test (B-SEVLT) assessed episodic learning and memory [23]. A 15-item list was presented for three consecutive learning trials followed by a 15-item distractor list and a memory trial immediately following the distractor list [24, 25]. Variables of interest included total learning across all three trials (range = 0–45) and total recall post-interference (i.e., memory; range = 0–15). The 2-letter Fluency test required participants to generate as many words as possible within 60 seconds that began with the letters ‘F’ and ‘A’ [26, 27]. The total number of correctly generated words summed across letters (range = 0–50) reflected executive functions of establishing/maintaining mental set and retrieval flexibility. The Digit Symbol Substitution Test (DSST) of the Wechsler Adult Intelligence Scale-Revised [28] required rapid copying and encoding of symbols to numbers within 90 seconds. The total number of correctly transcribed symbols during the time allotted (range = 0–80) denoted mental processing speed.

### Potential covariates

In addition to information on age, sex, education, and background, interviews obtained information on health insurance status (yes/no), income, marital status, and language preference for cognitive testing. Other measures of acculturation were quantified including length of US residence and social-based acculturation; however, they were highly correlated with language preference for cognitive testing and thus, not considered as additional covariates. We also evaluated overall mental status as measured by a brief mental status Six-Item Screener (SIS; higher = better performance) [29]. Additionally, depressive symptomatology was assessed with a modified, 10-item version of the Center for Epidemiologic Studies of Depression scale [30] (CESD-10; higher = more depressive symptoms).

Lastly, physical activity, known to affect cardiovascular and cognitive health [22], was evaluated using the World Health Organization Global Physical Activity Questionnaire [31] to determine levels of physical activity per the 2008 physical activity guidelines available at the time of the HCHS/SOL Visit 1 (http://www.health.gov/paguidelines/guidelines/default.aspx). Four mutually exclusive levels of physical activity were determined: inactive (no activity beyond baseline activities of daily living), low (activity beyond baseline but fewer than 150 minutes of moderate-intensity physical activity a week or the equivalent amount of vigorous-intensity activity or the equivalent combination of moderate and vigorous activity), medium (150 minutes to 300 minutes of moderate-intensity activity a week, or 75 to 150 minutes of vigorous-intensity physical activity a week, or the equivalent combination of moderate and vigorous activity), and high (more than the equivalent of 300 minutes of moderate-intensity physical activity a week, or more than 150 minutes of vigorous activity, or an equivalent combination of both) activity. Activity had to be performed in episodes of at least 10 minutes.

### Statistical analyses

All analyses were executed in SAS 9.3 (SAS Institute, Cary, NC) and accounted for the Hispanic Community Health Study/Study of Latinos (HCHS/SOL) sample design including sampling weights to allow appropriate generalizations to the target population, cluster sampling and stratification [18]. Descriptive statistics were compared across CVD-RF burden groups separately. Formal comparisons were carried out via overall survey-adjusted Wald tests. Results of these analyses helped determine relevant covariates. For continuous responses, all means and prevalence estimates were calculated using survey linear regression. Separate multivariable linear regressions were used to adjust for potential confounders. Statistical significance was defined as p<0.05.

Cognitive outcomes were deemed normal based on Q-Q plots. For each outcome, we fit two survey linear regression models: Model 1 included CVD-RF burden group as the predictor variable adjusting for age only. Model 2 additionally adjusted for sex, education, health insurance status, income, marital status, language preference during testing, SIS, CESD-10, and Hispanic/Latino background. In order to investigate whether Hispanic/Latino background was an effect modifier of the association between CVD-RF burden group and cognition, we added the interaction term of background*burden to each model (separate from the age models). We set significance for the interaction term to p≤0.05; as indicated, we conducted follow-up analyses to determine the background*burden categories driving significant relationships.

## Results

The lowest-burden group had 2,759 participants, the middle-burden group had 2,499 participants, and the highest-burden group had 1,827 participants. Table 1 shows the prevalence rates of individual CVD-RFs by burden group. Descriptive characteristics were compared across the three CVD-RF burden groups (Table 2). Groups differed on age (i.e., the lowest-burden group was youngest), education (e.g., the highest-burden group had disproportionately fewer individuals with more than a high school education), health insurance (i.e., the highest-burden group had a higher percentage of insured individuals than the lowest-burden group), income (i.e., the highest-burden group had fewer individuals making more than $50,000 than the lowest-burden group), and SIS (i.e., the highest-burden group scored the lowest). Hispanic/Latino background also differed, however, no other characteristics were significantly different across CVD-RF burden groups (Table 2). Thus, we included age, education, health insurance status, income, Hispanic/Latino background, and physical activity as covariates in fully-adjusted models. We also added terms for sex and language preference during testing given that these variables are known to be associated not only with our predictor variable but also our outcome variables.

**Table 1:** Percent composition of individual risk factors for all participants and by CVD-RF burden group.

	All Participants N = 7121	Lowest (0–2) n = 2795	Middle (3) n = 2499	Highest (4 or 5) n = 1827
Hypertension^a^	41.1 [39.2, 43.0]	9.7 [8.2, 11.3]	37.8 (34.6, 41.0)	86.6 (84.6, 88.5)
*anti-hypertensive medication*	25.1 [23.3, 26.8]	4.5 [3.4, 5.5]	20.7 (18.2, 23.2)	57.6 (53.7, 61.4)
Impaired Fasting Glucose (prediabetes) and Diabetes^b^	68.8 [67.2, 70.4]	34.5 [31.9, 37.1]	82.2 (80.0, 84.4)	97.6 (96.7, 98.5)
*anti-diabetes medication*	14.6 [13.0, 16.1]	3.6 [2.6, 4.6]	13.9 (11.9, 15.9)	29.8 (26.0, 33.6)
Hypercholesterolemia^c^	67.7 [65.9, 69.4]	39.7 [36.7, 42.8]	74.8 (72.1, 77.4)	95.8 (94.5, 97.2)
*anti-cholesterolemia medication*	17.1 [15.6, 18.6]	4.6 [3.3, 5.8]	13.9 (11.9, 15.9)	37.5 (33.9, 41.2)
Overweight or Obese^d^	83.3 [82.1, 84.6]	66.6 [64.0, 69.2]	89.4 (87.6, 91.1)	98.0 (97.1, 99.0)
Current Smoking^e^	18.9 [17.4, 20.4]	11.8 [10.0, 13.5]	15.8 (13.8, 17.9)	32.1 (28.8, 35.4)

Values represent the weighted mean percent [95% confidence intervals]; CVD-RF = cardiovascular disease risk factor.

^a^BP≥140/90 mmHg and/or on antihypertensive medication

^b^glucose≥100mg/dL or hemoglobin A1c≥5.7% and/or on medication for diabetes

^c^fasting total cholesterol ≥200mg/dL and/or use of cholesterol lowering medications

^d^BMI≥25kg/m^2^

^e^positive self-report of current smoking

**Table 2:** Characteristics for all participants and by CVD-RF burden group.

		All Participants N = 7121	Lowest (0–2) n = 2795	Middle (3) n = 2499	Highest (4 or 5) n = 1827	p-value
**Age**, years		55.9 [55.6, 56.2]	53.5 [53.0, 53.9]	56.2 [55.7, 56.7]	58.7 [58.1, 59.3]	<0.001
**Female**		54.7 [53.0, 56.4]	55.0 [52.4, 57.7]	55.4 [52.4, 58.4]	53.5 [50.0, 57.0]	0.72
**Education**						
< High School		38.1 [36.0, 40.3]	34.3 [31.2, 37.3]	40.0 [36.2, 43.7]	41.0 [37.6, 44.3]	0.0038
High School graduate		21.9 [20.2, 23.5]	21.8 [19.5, 24.1]	20.4 [17.8, 23.1]	23.7 [20.6, 26.8]	0.2736
Greater than high school		40.0 [38.1, 42.0]	43.9 [41.1, 46.8]	39.6 [36.3, 42.9]	35.3 [31.7, 38.9]	<0.001
**Health Insurance**		53.7 [51.0, 56.3]	49.6 [46.1, 53.0]	53.3 [50.0, 56.5]	59.5 [55.5, 63.5]	<0.001
**Annual Family Income**						
<$20,000		43.2 [40.9, 45.5]	42.6 [39.4, 45.9]	41.6 [38.4, 44.8]	45.8 [41.8, 49.8]	0.19
$20,000–50,000		36.7 [34.8, 38.6]	37.8 [34.8, 40.7]	36.3 [33.4, 39.1]	35.7 [31.8, 39.6]	0.63
>50,000		11.9 [10.1, 13.8]	12.5 [10.1, 15.0]	13.8 [10.4, 17.1]	8.9 [6.9, 10.8]	0.002
Not reported		8.2 [7.3, 9.2]	7.1 [5.8, 8.4]	8.3 [6.6, 10.0]	9.6 [7.8, 11.4]	0.052
**Marital Status**						
Single		15.5 [14.2, 16.8]	15.7 [13.9, 17.4]	14.6 [12.4, 16.9]	16.3 [13.9, 18.7]	0.56
Married or living with a partner		56.0 [53.7, 58.4]	57.4 [54.0, 60.7]	57.6 [54.4, 60.9]	52.3 [48.2, 56.4]	0.06
Separated, divorced or widowed		28.5 [26.5, 30.4]	27.0 [24.0, 30.0]	27.7 [25.0, 30.5]	31.4 [27.6, 35.2]	0.16
**Language Preference (Spanish)**	86.5 [84.7, 88.3]		84.6 [81.8, 87.4]	88.1 [85.8, 90.4]	87.0 [83.8, 90.2]	0.12
**Six Item Screener score**	5.3 [5.3, 5.4]		5.4 [5.4, 5.4]	5.4 [5.3, 5.4]	5.3 [5.2, 5.3]	0.01
**10-item CESD score**	6.9 [6.6, 7.1]		6.7 [6.2, 7.1]	6.8 [6.5, 7.2]	7.2 [6.7, 7.6]	0.25
**Hispanic/Latino background**						
Mexican (n = 2,836)		33.8 [30.0, 37.5]	35.0 [31.1, 38.9]	38.5 [33.6, 43.4]	26.4 [21.3, 31.5]	<0.001
Cuban (n = 1,110)		25.6 [21.6, 29.5]	23.0 [18.7, 27.3]	23.8 [19.5, 28.0]	31.1 [25.9, 36.4]	<0.001
Puerto Rican (n = 1,142)		16.4 [14.4, 18.5]	15.7 [12.6, 18.8]	14.1 [11.8, 16.4]	20.2 [16.6, 23.7]	0.005
Dominican (n = 597)		8.7 [7.3, 10.1]	9.5 [7.4, 11.5]	7.8 [6.0, 9.5]	8.9 [7.2, 10.6]	0.20
Central American (n = 749)		6.9 [6.0, 7.9]	7.2 [5.8, 8.6]	7.9 [6.4, 9.3]	5.5 [4.4, 6.6]	0.01
South American (n = 542)		6.2 [5.4, 7.0]	7.6 [6.3, 8.9]	5.7 [4.4, 6.9]	5.0 [3.7, 6.3]	0.008
Other (n = 145)		2.4 [1.6, 3.2]	2.1 [1.4, 2.8]	2.4 [1.5, 3.2]	2.8 [0.6, 5.0]	0.73
**Physical Activity Level**						
Inactive		26.3 [24.5, 28.1]	24.0 [21.3, 26.7]	25.5 [22.5, 28.6]	30.1 [26.8, 33.5]	<0.001
Low		14.4 [13.0, 15.7]	13.6][11.7, 15.5]	14.1 [11.5, 16.8]	15.6 [13.5, 17.7]	<0.001
Medium		11.1 [10.1, 12.2]	11.7 [9.9, 13.5]	10.1 [8.7, 11.6]	11.7 [9.8, 13.6]	<0.001
High		48.2 [46.4, 50.1]	50.7 [47.7, 53.8]	50.2 [47.2, 53.3]	42.6 [39.0, 46.1]	<0.001
**Other measures of acculturation**						
US residence >10y %		74.9 [72.5, 77.4]	74.3 [71.3, 77.2]	75.8 [72.8, 78.7]	74.8 [70.7, 78.9]	0.70
Social-based acculturation score		2.2 [2.1, 2.2]	2.2 [2.2, 2.2]	2.2 [2.1, 2.2]	2.1 [2.1, 2.2]	0.10
***Cognitive Test Performance***						
*B-SEVLT Learning*		22.8 [22.6, 23.1]	23.5 [23.2, 23.9]	22.8 [22.5, 23.2]	21.9 [21.5, 22.3]	<0.001
*B-SEVLT Recall*		8.3 [8.2, 8.4]	8.7 [8.5, 8.8]	8.2 [8.1, 8.4]	7.8 [7.6, 8.0]	<0.001
*2-Letter Fluency*		18.7 [18.4, 19.0]	19.4 [18.9, 19.8]	18.6 [18.0, 19.2]	17.8 [17.3, 18.3]	<0.001
*Digit Symbol Substitution*		34.6 [33.9, 35.2]	36.9 [36.1, 37.7]	34.3 [33.4, 35.2]	31.9 [30.7, 33.0]	<0.001

Values represent the weighted mean percent [95% confidence intervals] unless otherwise noted; CVD-RF = cardiovascular risk factor. Cognitive test score ranges were as follows: B-SEVLT Learning = 0–45, Recall = 0–15, 2-Letter Fluency = 0–50, Digit Symbol Substitution = 0–80.

### CVD-RF burden and cognition

The highest burden group had significantly lower scores (i.e., on average, ½ point to 2-points lower, p-values<0.001) on all cognitive tests except letter fluency compared to the lowest burden group in age-adjusted Model 1 (Table 3). Only scores for learning (Beta = -0.55, 95% CI [-1.06, -0.04], p<0.05) differed between the highest and the middle burden group after age-adjustment. In contrast, age-adjusted analyses revealed that scores for total recall post-interference and letter fluency were significantly different between the middle- and lowest-burden groups (p-values <0.05; Model 1, Table 3).

**Table 3:** Association of CVD-RF burden groups with cognition.

		Cognitive Test Scores			
Model	Burden Group	B-SEVLT Learning Beta [95% CI]	B-SEVLT Recall Beta [95% CI]	2-Letter Fluency Beta [95% CI]	Digit Symbol Substitution Beta [95% CI]
1					
	Middle vs lowest	-0.27 [-0.70, 0.17]	-0.21 [-0.43, -0.00]^a^	-0.64 [-1.39, -0.11]^c^	-0.99 [-2.03, 0.04]
	Highest vs lowest	-0.82 [-1.32, -0.31]^b^	-0.42 [-0.66, -0.17]^c^	-1.31 [-1.90, -0.71]	-1.95 [-3.11, -0.80]^b^
	Highest vs middle	-0.55 [-1.06, -0.04]^a^	-0.20 [-0.47, 0.07]	-0.67 [-1.42, 0.09]	-0.96 [-2.26, 0.34]
2					
	Middle vs lowest	-0.26 [-0.66, 0.14]	-0.24 [-0.43, -0.04] ^a^	-0.59 [-1.21, 0.03]	-0.70 [-1.50, 0.10]
	Highest vs lowest	-0.43 [-0.89, 0.03]	-0.21 [-0.44, 0.02]	-0.86 [-1.45, -0.27]^b^	-1.26 [-2.15, -0.36]^b^
	Highest vs middle	-0.17 [-0.65, 0.30]	0.03 [-0.23, 0.28]	-0.27 [-0.94, 0.41]	-0.56 [-1.56, 0.44]

Sample sizes by Burden Group: Lowest (n = 2,795), Middle (n = 2,499), Highest (n = 1,827)

CVD-RF = cardiovascular risk factor, Beta = unstandardized regression coefficient, CI = confidence interval. B-SEVLT = Brief Spanish English Verbal Learning Test; Highest-burden group = any 4 or 5 risk factors, middle-burden group = any 3 risk factors, lowest-burden group = any 0–2 risk factors. Cognitive test score ranges were as follows: B-SEVLT Learning = 0–45, Recall = 0–15, 2-Letter Fluency = 0–50, Digit Symbol Substitution = 0–80.

Model 1: Adjusted for age only

Model 2: Adjusted for Age as well as sex, education, health insurance status, income, language preference during testing, Hispanic/Latino background, and physical activity

^a^p<0.05

^b^p<0.01

^c^p<0.001

After additional adjustment for sex, education, health insurance status, income, language preference during testing, Hispanic/Latino background, and physical activity (Model 2, Table 3), the highest burden group continued to be associated with lower digit symbol substitution performance by an average of 1 point when compared to the lowest-burden group, p<0.05. Additionally, the highest burden group became associated with lower letter fluency also compared to the lowest-burden group (Beta = -0.80, 95% CI [-1.39, -0.21], p<0.01). Lastly, the middle-burden group continued to differ from the lowest burden group on total recall post-interference (Beta = -0.22, 95% CI [-0.41, -0.02], p<0.05).

### CVD-RF burden, cognition, and background

When considering Hispanic/Latino background, only the background*burden interaction for learning and recall post-interference was significant in fully adjusted models (p-values≤0.03). That is, background differences (e.g., Mexicans vs Cuban) depend on the burden level (i.e., lowest, middle, or highest). As seen in Tables 4 and 5, when compared to participants reporting a Mexican background in the lowest CVD-RF burden group, participants reporting a Cuban or Puerto Rican background in the lowest CVD-RF burden group had lower B-SEVLT learning and recall scores while participants reporting a Dominican background in this same (lowest) CVD-RF burden group had lower B-SEVLT recall scores only. When compared to participants reporting a Mexican background in the middle CVD-RF burden group, participants reporting either a Puerto Rican background in this same (middle) CVD-RF burden group had lower B-SEVLT learning (Table 4) and recall (Table 5) scores while participants reporting a Cuban or Dominican background in the middle CVD-RF burden group had lower B-SEVLT recall scores only (Table 5). Lastly, compared to participants reporting a Mexican background in the highest CVD-RF burden group, participants reporting a Cuban, Puerto Rican, or Dominican background also in the highest CVD-RF burden group had lower B-SEVLT learning and recall scores while participants reporting a Central American background in the highest CVD-RF burden group had lower B-SEVLT recall scores only (Tables 4 and 5).

**Table 4:** Follow-up comparisons of significant model 2 background*burden associations with B-SEVLT learning.

	CVD-RF Burden Groups		
Background	Lowest (0–2) Beta [95% CI]	Middle (3) Beta [95% CI]	Highest (4 or 5) Beta [95% CI]
Mexican (n = 2,836)	REF	REF	REF
Cuban (n = 1,110)	**-1.47** **[-2.24, -0.69]**	-0.82 [-1.68, 0.04]	**-1.55** **[-2.49, -0.61]**
Puerto Rican (n = 1,142)	**-1.34** **[-2.42, -0.26]**	**-1.77** **[-2.71, -0.83]**	**-2.29** **[-3.13, -1.45]**
Dominican (n = 597)	-0.23 [-1.07, 0.60]	0.15 [-0.91, 1.22]	**-1.54** **[-2.56, -0.52]**
Central American (n = 749)	-0.78 [-1.58, 0.02]	0.25 [-0.66, 1.16]	**-1.12** **[-2.24, -0.01]**
South American (n = 542)	0.15 [-0.69, 0.99]	0.07 [-1.10,1.23]	-0.09 [-1.62,1.44]

Sample sizes for Background by Burden Group: Mexican—Lowest (n = 1,168), Middle (n = 1,064), Highest (n = 604); Cuban—Lowest (n = 397), Middle (n = 359), Highest (n = 354); Puerto Rican—Lowest (n = 377), Middle (n = 375), Highest (n = 390); Dominican—Lowest (n = 241), Middle (n = 188), Highest (n = 168); Central American—Lowest (n = 288), Middle (n = 296), Highest (n = 165); South American—Lowest (n = 268), Middle (n = 167), Highest (n = 107)

CVD-RF = cardiovascular risk factor, Beta = unstandardized regression coefficient, CI = confidence interval. Highest-burden group = any 4 or 5 risk factors, middle-burden group = any 3 risk factors, lowest-burden group = any 0–2 risk factors. B-SEVLT Learning test score ranged from 0–45. Analyses adjusted for age, sex, education, health insurance status, income, language preference during testing, CESD-10, Hispanic/Latino background, and physical activity. Bolded entries indicate confidence intervals that do not cross zero.

**Table 5:** Follow-up comparisons of significant model 2 background*burden associations with B-SEVLT recall.

	CVD-RF Burden Groups		
Background	Lowest (0–2) Beta [95% CI]	Middle (3) Beta [95% CI]	Highest (4 or 5) Beta [95% CI]
Mexican (n = 2,836)	REF	REF	REF
Cuban (n = 1,110)	**-0.84** **[-1.16,-0.52]**	**-0.55** **[-0.95,-0.15]**	**-0.80** **[-1.27,-0.33]**
Puerto Rican (n = 1,142)	**-0.87** **[-1.28, -0.47]**	**-1.55** **[-2.04, -1.06]**	**-1.61** **[-2.12, -1.11]**
Dominican (n = 597)	**-0.80** **[-1.31, -0.29]**	**-0.92** **[-1.58, -0.27]**	**-1.28** **[-1.82, -0.74]**
Central American (n = 749)	-0.38 [-0.80, 0.04]	0.11 [-0.34, 0.56]	-0.37 [-0.95, 0.21]
South American (n = 542)	-0.21 [-0.60, 0.18]	-0.36 [-0.95, 0.23]	0.25 [-0.54, 1.05]

Sample sizes for Background by Burden Group: Mexican—Lowest (n = 1,168), Middle (n = 1,064), Highest (n = 604); Cuban—Lowest (n = 397), Middle (n = 359), Highest (n = 354); Puerto Rican—Lowest (n = 377), Middle (n = 375), Highest (n = 390); Dominican—Lowest (n = 241), Middle (n = 188), Highest (n = 168); Central American—Lowest (n = 288), Middle (n = 296), Highest (n = 165); South American—Lowest (n = 268), Middle (n = 167), Highest (n = 107)

CVD-RF = cardiovascular risk factor, Beta = unstandardized regression coefficient, CI = confidence interval. Highest-burden group = any 4 or 5 risk factors, middle-burden group = any 3 risk factors, lowest-burden group = any 0–2 risk factors. B-SEVLT Recall test score ranged from 0–15. Analyses adjusted for age, sex, education, health insurance status, income, language preference during testing, CESD-10, Hispanic/Latino background, and physical activity. Bolded entries indicate confidence intervals that do not cross zero.

While we did not have adequate sample size distributions of participants by background to stratify by individual CVD-RFs that comprised our burden score to formally probe which CVD-RF may have driven the above reported background*burden distinctions, we did review individual CVD-RF profiles by these backgrounds. Of the 5 CVD-RFs measured for the background groups showing the most consistent differences from the reference group (i.e., Cuban, Puerto Rican, and Dominican backgrounds compared to Mexicans), hypertension was most prevalent (43–48%). Puerto Ricans had one of the highest percentages of combined impaired fasting glucose and/or diabetes (72%) while Cubans had one of the highest percentages of hypercholesterolemia compared to all other Hispanic/Latino backgrounds. Lastly, participants reporting a Cuban or Puerto Rican background had the highest percentages of current smoking (26%) compared to all other Hispanic/Latino backgrounds (10–17%).

## Discussion

We identified associations between higher CVD-RF burden and lower cognition in Hispanics/Latinos generally and by self-reported background specifically. Hispanics/Latinos at the highest levels of CVD-RF burden performed worse than Hispanics/Latinos at the lowest levels of CVD-RF burden on a task of mental processing speed and a measure of fluency in a fully-adjusted models. Hispanic/Latino background modified the effect of burden on learning and post-interference recall with participants from a Cuban, Puerto Rican, or Dominican background with the lowest, middle, and/or highest CVD-RF burden showing reduced cognitive functioning when compared to their Mexican counterparts in the identical CVD-RF burden groups. Thus, it is important to consider not only CVD-RF burden when evaluating cognitive functioning in Hispanics/Latinos but also self-reported background, particularly when using normative data that may or may not represent that individual’s background, let alone ethnicity.

Underlying mechanisms to explain associations reported in the overall sample, while beyond the scope of this study, may be found in the patterns of individual CVD-RFs seen in study participants, the larger HCHS/SOL cohort [6, 15], and in the implications for these individual risk factors as it relates to cognition and brain aging found in the literature. For example, what distinguished the highest burden group from the middle and lower groups (independent of background) was the presence of hypertension. Hypertension is known to negatively impact cerebral perfusion [32] and in turn, grey [32] and white [33] matter integrity, particularly within frontal and temporal regions [34]. When combined with other CVD-RFs, this may have led to lower performance on measures of mental processing speed and set maintenance when compared to the other burden groups. In contrast, the middle burden group who performed worse than the lower burden group on memory testing showed a pattern of increased diabetes, hypercholesterolemia, and obesity. This triad of risk is often seen in association with metabolic syndrome [35], a condition known to negatively impact learning and memory in affected individuals (e.g., [10]). Thus, the selectivity of our cognitive results may reflect the unique patterns of risk seen by CVD-RF groups generally, however, explanations for results by Hispanic/Latino background require consideration of why CVD-RF burden may have more of an impact in one group than another.

Results of this study suggests that a closer understanding of self-reported background and the implications for level of CVD-RF burden are warranted when considering Hispanic/Latino participants’ cognitive test results with our observations of individual CVD-RFs by background in this study, and the larger HCHS/SOL [6, 15] suggesting potential reasons for these distinctions. Follow-up analyses suggested a linear relationship between increasing CVD-RF burden and decreasing memory for Puerto Ricans and Dominicans when compared to their Mexican counterparts with similar levels of CVD-RF burden. A similar pattern of association was seen between CVD-RF burden and learning only for Puerto Ricans across CVD-RF groups compared to Mexicans across similar groups. Contrary to our hypothesis, Cubans showed a pattern of associations between CVD-RF burden and cognition such that Cubans in the lowest and highest burden groups showed the most reduced scores on learning and memory when compared to their Mexican counterparts of respective burden. Lastly, Dominicans and Central Americans in the highest burden groups displayed lower memory scores compared to Mexicans in the same burden group. A review of individual CVD-RF profiles by background suggests that CVD-RF burden may have more of an impact in participants from specific backgrounds, i.e., Puerto Rican and to a lesser extent Cuban and Dominican, than other Hispanic/Latino backgrounds. Generally speaking, overall burden in these groups is higher than in Mexicans [6, 15], placing these individuals at a disadvantage compared to the reference group regardless of outcome. More specifically, the combination of hypertension, smoking, and an additional CVD-RF (e.g., impaired fasting glucose and/or diabetes for Puerto Ricans) may have a particularly robust relationship with cognition in these Hispanic/Latino backgrounds. These observed distinctions in individual CVD-RF burden profiles should be the focus of future directed studies of cognition by backgrounds with enough power to investigate information regarding CVD-RF severity and duration and fully understand the interaction of background by burden in Hispanics/Latinos.

Our consideration of the role of self-reported background on the relationship between CVD-RF burden and cognition is, to our knowledge, one of the first reports of its kind suggesting differential associations based on Hispanic/Latino background. Our work investigating the relationship between CVD-RF burden and cognition in mid- to late-life Hispanics/Latinos adds to the existing literature in additional ways. The overall results are consistent with other studies (e.g., [8]) involving Hispanics/Latinos that report a relationship between higher cumulative CVD-RF burden and lower mental processing speed, fluency, and memory. In contrast to other studies [8, 22, 36, 37], we did not find a consistent linear association between increasing CVD-RF burden and decreasing cognitive test performance, only a distinction in performance based on a comparison of the highest and lowest burden groups. This may be due, in part, to the fact that we investigated multiple CVD-RFs while previous work focused on individual CVD-RFs (e.g., diabetes: [36, 37]) or multiple CVD-RFs plus lifestyle factors (e.g., [22]). Additionally, our study extends previous work in HCHS/SOL [17] reporting that Hispanics/Latinos of Mexican background showed the highest levels of cognitive performance compared to all other backgrounds, by revealing that this result is also evident when compared to select background groups at varying levels of CVD-RF burden.

Across all results of this study, the statistically significant associations between CVD-RF burden and cognition and the test point decrements they represent may not equate to clinically significant cross-sectional implications. They may, however, represent harbingers of accelerated cognitive decline in later life given the relatively young age of our cohort. For example, while a 1–2 point difference in cognitive testing may not meet standard cut-points for at-risk states for dementia including mild cognitive (MCI) or vascular cognitive impairment (VCI) [38, 39], it may place Hispanic/Latino individuals with multiple CVD-RFs at increased risk for accelerated aging by moving his/her scores closer to these cut-points compared to an individual with fewer CVD-RFs [40]. In fact, a recent study of cognitively normal individuals with two or more chronic conditions (including CVD-RFs) found that after a median 4-year follow up they had a higher risk of developing MCI [41]. Although older adults in our study displayed the higher burden/lower cognitive performance profile whereas younger adults did not, this and other studies suggest that cross-sectional associations between higher burden and lower cognition emerge in Hispanics/Latinos by the sixth decade of life. Thus, cumulative CVD-RF burden may have implications for the earlier age of dementia diagnosis in this population compared to non-Hispanic whites [42–44]. Ongoing longitudinal follow up in HCHS/SOL will be invaluable in determining the role of baseline CVD-RF burden in cognitive decline in mid- to late-life Hispanics/Latinos.

As with any cross-sectional study, the direction and causes of our CVD-RF/cognitive test associates cannot be explicitly known. Furthermore, our summary of CVD-RF burden should not be taken as an indication that all CVD-RFs associate with cognitive testing in the same manner, e.g., severity of individual CVD-RFs may differentially affect cognition. Despite this, we contend that the number of CVD-RFs increases with age and are rarely found in isolation in Hispanics/Latinos [6]. Thus, the relationship between composite CVD-RF burden and cognitive testing should not be discounted in this population. We did not have access to participants’ medical records, thus, we were unable to determine duration of each CVD-RF, or confirm self-reported presence/absence of stroke; however, we did verify antipsychotic medication use and all other CVD-RF medication use at study visit through medication review. While select CVD-RFs have been studied in terms of treatment-related controlled, e.g., uncontrolled hypertension has been shown to negatively impact learning and memory in Hispanics/Latinos (e.g., [45]), less is known about the best approach to modeling the role of treatment-related control in a CVD-RF burden approach that incorporates multiple risk factors, some, but not all of which may be treated pharmacologically and which may contribute to a diagnosis of a particular CVD-RF. Thus, as with any study, not all potential modifiers could be considered or confounders for that matter. For example, we did not adjust for Stage IV kidney disease, present in 0.5% of our study participants, hemodialysis, head injury, or Parkinson’s disease. Additionally, information on nutritional differences by background, not adjusted for in the current project, may represent an additional contributor to our findings. Lastly, given that the focus of the HCHS/SOL study was cardiovascular in nature [19], our cognitive testing was limited; however, it incorporated important cognitive outcomes associated with aging including learning, memory, and attention/executive functioning. Thus, results of this study should be interpreted with caution and within the context of these limitations.

To conclude, prior reports suggest that 80% of Hispanic/Latino men and 71% of Hispanic/Latina women in HCHS/SOL have at least one CVD-RF regardless of background and roughly 30–40% of this same population report greater CVD-RF burden [6]. This equates to a 25–30% higher prevalence of at least one CVD-RF, e.g., hypertension, compared to non-Hispanic Blacks and a 40–50% higher prevalence compared to non-Hispanic Whites [46]. This suggests, and our results supports, that cumulative CVD-RF burden may have implications for specific aspects of cognition in Hispanics/Latinos, and may place Hispanics/Latinos at increased risk of accelerated aging as early as the 5^th^ and 6^th^ decades of life. Additionally, our results suggest that levels of CVD-RF burden may be particularly detrimental to cognition for individuals from select backgrounds when compared to their Mexican counterparts at equal levels of CVD-RF burden. Thus, more work is needed to understand not just the cross-sectional associations but also the longitudinal changes in cognitive performance related to health disparities in CVD-RFs disproportionately affecting Hispanics/Latinos generally [47], and by background specifically [2].
